# Diversity and Biosynthetic Potential of Culturable Microbes Associated with Toxic Marine Animals

**DOI:** 10.3390/md11082695

**Published:** 2013-08-02

**Authors:** Rocky Chau, John A. Kalaitzis, Susanna A. Wood, Brett A. Neilan

**Affiliations:** 1 School of Biotechnology and Biomolecular Sciences, The University of New South Wales, Sydney, NSW 2052, Australia; E-Mails: rocky.chau@student.unsw.edu.au (R.C.); jkalaitzis@unsw.edu.au (J.A.K.); 2 Cawthron Institute, Private Bag 2, Nelson, 7001 and Department of Biological Sciences, University of Waikato, Private Bag 3105, Hamilton 3240, New Zealand; E-Mail: susie.wood@cawthron.org.nz

**Keywords:** tetrodotoxin, biosynthesis, microbial diversity, *Pleurobranchaea maculata*, *Hapalochlaena* sp., *Nassarius semiplicatus*

## Abstract

Tetrodotoxin (TTX) is a neurotoxin that has been reported from taxonomically diverse organisms across 14 different phyla. The biogenic origin of tetrodotoxin is still disputed, however, TTX biosynthesis by host-associated bacteria has been reported. An investigation into the culturable microbial populations from the TTX-associated blue-ringed octopus *Hapalochlaena* sp. and sea slug *Pleurobranchaea maculata* revealed a surprisingly high microbial diversity. Although TTX was not detected among the cultured isolates, PCR screening identifiedsome natural product biosynthesis genes putatively involved in its assembly. This study is the first to report on the microbial diversity of culturable communities from *H. maculosa* and *P. maculata* and common natural product biosynthesis genes from their microbiota. We also reassess the production of TTX reported from three bacterial strains isolated from the TTX-containing gastropod *Nassarius semiplicatus*.

## 1. Introduction

**Figure 1:**
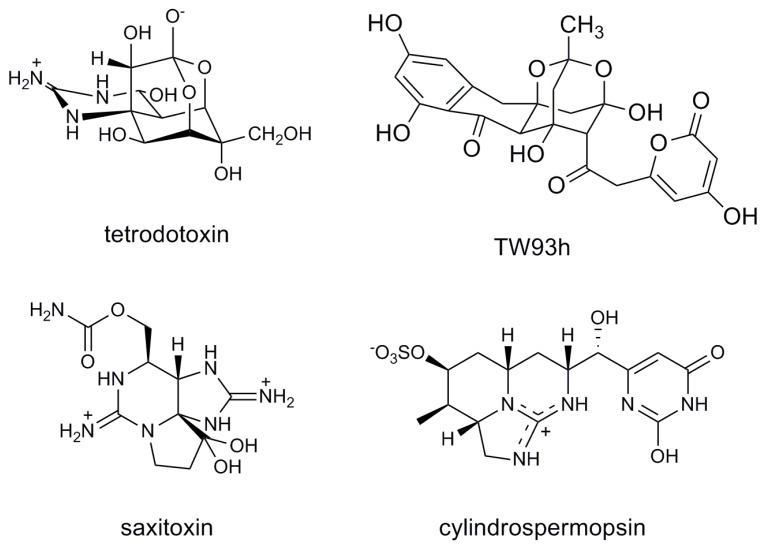
Structures of tetrodotoxin and structurally-related toxins from marine algae and cyanobacteria.

Soft-bodied marine organisms, particularly sessile or slow moving invertebrates, have evolved complex chemical defence systems to enhance their competitive fitness and, ultimately, survival [1]. Some of the common, but often complex, chemical defences serve to function as antifoulants, camouflaging agents, or UV-absorbing sunscreens. The same marine organisms possess little or no means of physical defence against predation and therefore aggressively combat predators with lethal toxins. The production of these toxins by such marine invertebrates is one of the most intriguing and intensely studied facets of natural chemical defence systems. The focus of this study is one of the better known, marine toxins, tetrodotoxin (TTX; Figure 1). 

TTX is one of the most toxic natural substances known and symptoms of human TTX poisoning include numbness of the face and extremities, paralysis, respiratory failure, circulatory collapse, and death [2]. TTX is a sodium channel blocker and some TTX-bearing organisms, including *Taricha* newts and *Tetraodontidae* fish, nullify the auto-effect of the toxin by possessing TTX-resistant sodium channels [3] while other species, for example the shore crab *Hemigrapsus sanguineus*, have been found to produce TTX-binding compounds that are able to neutralize the effects of TTX [4]. 

TTX has been reported from taxonomically diverse organisms across 14 different phyla [5], including puffer fish from the family *Tetraodontidae* from which it takes its name, the blue-ringed octopus *Hapalochlaena maculosa* [6], the grey side-gilled sea slug *Pleurobranchaea maculate* [7], and marine gastropod *Nassarius semiplicatus* [8]. The widespread occurrence strongly suggests that microorganisms are the true source of TTX in nature. It is unlikely that the complex biosynthetic machinery, responsible for TTX production, would have co-evolved in such numerous phylogenetically and environmentally diverse higher organisms and thus it has been proposed that TTX’s broad distribution may be due to horizontal transfer, via acquisition through the food chain, of TTX-producing bacteria [9,10]. This hypothesis is supported by studies in which TTX-producing bacteria have been isolated from many different host organisms (reviewed in Chau *et al.*, 2011 [5]). 

TTX-producing bacteria belonging to the genera *Vibrio*, *Bacillus*, and *Pseudomonas* have been isolated from numerous organisms including blue-ringed octopus (*Hapalochlaena* sp.), puffer fish (Fugu spp.), and deep-sea sediments [5]. Numerous studies have identified multiple species of toxic bacteria from a single host organism, suggesting that TTX-producing bacteria are abundant in these organisms. Twenty TTX-producing bacterial strains from at least two genera have been isolated from the common puffer fish *Fugurubripes* [11]. Similarly, 21 TTX-producing bacterial strains belonging to five genera have been isolated from the gastropod *N. semiplicatus* [8]. It is pertinent to note that most of these studies were performed prior to the 1990s, and thus 16S ribosomal RNA gene sequence analysis was not used as a tool for determining bacterial taxonomy and phylogeny, as it is today. Because of the inconsistencies thatare often encountered when classifying microorganisms using a polyphasic approach, there is an urgent need to reassess microbial communities derived from TTX-containing host organisms.

Despite evidence of the bacterial production, some argument over TTX’s origin still exists. While TTX has been isolated from bacteria in some animals, it has been reported that some TTX associated organisms, such as *Tarichatorosa*, do not harbor toxic endosymbionts [12]. 

Modern molecular tools have been developed that enable the identification of a microorganism’s genetic potential to biosynthesize discrete small bioactive molecules. Ultimate proof of TTX’s proposed microbial origin, or indeed that of any microbial natural product, can be achieved by identifying biosynthesis genes involved in its assembly. However, in the case of TTX, its biosynthetic pathway remains elusive, as does a stable and reliable microbial producer of the molecule. A number of TTX biosynthesis pathway proposals have been documented [13,14,15], however, no supporting or experimental evidence has been published. In addition, biosynthetic feeding studies incorporating ^14^C-labeled acetate and guanido-^14^C labeled arginine have failed to reveal the mechanism or pathway for TTX biosynthesis [16]. A focus of our research is to understand the biosynthesis of small, naturally occurring toxins by microorganisms at the molecular level. No conclusive evidence has been published for the biosynthesis of TTX, however, based on our understanding of biosynthesis gene clusters of guanidine-containing toxins saxitoxin and cylindrospermopsin and the dioxoadamantane-containing polyketide, TW93h [17,18,19] (Figure 1), we speculate that TTX is assembled by a hybrid polyketide synthase (PKS)/non-ribosomal peptide synthetase (NRPS) enzyme complex which possibly incorporates an amidinotransferase (AMT) [5]. The genes encoding TTX biosynthesis enzymes along with those associated with toxin regulation and transport are proposed to be clustered on a single genome, in a similar manner to other toxin biosynthesis pathways in bacteria. This study describes the initial isolation of microbial populations from two TTX-associated host organisms, *Hapalochlaena* sp. and *P. maculata*. Additionally, LC-MS methods were used to reassess the production of TTX from *N. semiplicatus* bacterial isolates that had been previously identified as TTX-producers using immunoassay-based methods [8].

In order to identify biosynthesis genes putatively involved in small molecule assembly, including TTX, we mined the genomes of a library of diverse bacteria, isolated from these organisms, using a degenerate PCR-based screening approach. Here we present the outcomes of our genome mining study and report on the diversity of the culturable microbial communities associated with these hosts.

## 2. Results and Discussion

### 2.1. Bacterial Diversity in Toxic *Hapalochlaena* sp. and *Pleurobranchaea maculata*

A total of 27 and 22 unique bacterial isolates were cultured from *Hapalochlaena* sp. and *P*. *maculata*, respectively. In order to classify these isolates, approximately 1400 bp of the 16S rRNA gene was successfully amplified and sequenced from each isolate (GenBank accession numbers JN618116 to JN618164) and analyzed using the NCBI BLASTn algorithm. This revealed that *Alteromonadales* were predominant, with 30 of 49 isolates accounted for by this taxonomic order (Table 1, Table 2). *Pseudoalteromonas* and *Alteromonas* were the major representatives of *Alteromonadales* in both host organisms. 

**Table 1:** Bacterial identification and screening for polyketide synthase (PKS), non-ribosomal peptide synthetase (NRPS), and amidinotransferase (AMT) genes related to tetrodotoxin (TTX)-biosynthesis in bacterial isolates from *Hapalochlaena* sp.

Strain	Closest 16S rRNA gene BLAST match^a^	Identity	PCR screening			TTX
		(%)	PKS	NRPS	AMT	Detection^b^
HM BE02	*Alteromonadales* bacterium HOT3G5 (HQ537362.1)	99	-	-	-	-
HM BE03	*Alteromonas* sp. S2542 (FJ457277.1)	99	-	-	-	-
HM BE04	*Alteromonas* sp. S2542 (FJ457277.1)	99	-	-	-	-
HM BE05	*Pseudoalteromonas* sp. AmSamW21 (GU903211.1)	99	+	-	-	-
HM BE06	*Vibrio* *rotiferianus* isolate AP17 (HE584775.1)	99	-	-	-	-
HM LI01	*Nautellaitalica* strain R-28753 (AM944522.1)	99	-	-	+	NT
HM LI02	*Alteromonas* sp. S2542 (FJ457277.1)	99	-	-	-	-
HM LI03	*Pseudoalteromonadaceae* bacterium S3 (HQ164448.1)	100	-	-	-	NT
HM LI04	*Nautellaitalica* strain R-28753 (AM944522.1)	99	-	-	-	NT
HM LI05	*Alteromonas* sp. S2542 (FJ457277.1)	99	-	-	-	NT
HM LI06	*Pseudoalteromonas* sp. AKA07-4 (AB571944.1)	99	-	-	-	NT
HM OE02	*Pseudoalteromonadaceae* bacterium S3 (HQ164448.1)	100	-	-	+	NT
HM OE03	*Alteromonas* sp. CF14-3 (FJ170033.1)	99	-	-	-	-
HM OE04	*Thalassomonas* sp. PaD1.04 (GQ391976.1)	98	-	-	-	-
HM OE07	*Pseudoalteromonas* sp. TB51 (JF273853.1)	99	-	-	-	-
HM OE08	*Colwellia* sp. KMD002 (EU599214.3)	98	-	-	-	NT
HM OE09	*Pseudoalteromonadaceae* bacterium S3 (HQ164448.1)	99	+	-	+	NT
HM SA02	*Pseudoalteromonas* sp. AKA07-4 (AB571944.1)	99	+	+	+	-
HM SA03	*Pseudoalteromonadaceae* bacterium S3 (HQ164448.1)	99	+	+	+	-
HM SA04	*Pseudoalteromonas* sp. NW 4327 strain NW (FR839670.1)	99	+	-	-	NT
HM SA05	*Marinomonas* *communis* strain LMG 2864 (DQ011528.1)	99	-	-	-	NT
HM SA06	*Alteromonas* sp. KB19 (HM583350.1)	99	-	-	-	-

^a^ NCBI Genbank accession numbers are indicated in brackets; ^b^ NT indicates that the sample was not tested for its ability to produce TTX. The detection limit of TTX using the LC-MS method was 0.1 ng/mL.

**Table 2:** Bacterial identification and screening for polyketide synthase (PKS), non-ribosomal peptide synthetase (NRPS), and amidinotransferase (AMT) genes related to tetrodotoxin (TTX)-biosynthesis in bacterial isolates from *Pleurobranchaea maculata*.

Strain	Closest 16S rRNA gene BLAST match ^a^	Identity	PCR screening			TTX
		(%)	PKS	NRPS	AMT	Detection ^b^
PM DT05	*Photobacterium* * kishitanii* strain S-27 (JF412253.1)	99	-	-	-	-
PM DT08	*Shewanella* *schlegeliana* (AB081761.1)	99	-	-	-	-
PM DT10	*Photobacterium* sp. DFC2.17 isolate DFC2.17 (FR873783.1)	99	-	-	-	-
PM DT11	*Photobacterium* * kishitanii* strain S-27 (JF412253.1)	99	-	-	-	NT
PM DT12	*Shewanella* * schlegeliana* (AB081761.1)	99	+	-	-	-
PM EG02	*Vibrio* sp. YDO5 (GU586127.1)	98	-	-	-	-
PM EG04	*Staphylococcus warneri* strain Na58 (HQ831387.1)	99	-	-	-	-
PM EG05	*Staphylococcus warneri* strain Na59 (HQ831388.1)	99	-	-	-	-
PM EG08	*Pseudoalteromonas* sp. 114Z-11 (GU584139.1)	99	-	-	-	-
PM EG09	*Pseudoalteromonas* sp. BSs20138 (EU365489.1)	99	-	-	-	-
PM EG11	*Alteromonas* sp. N98(2010) (HQ188650.1)	99	-	+	-	-
PM EG13	*Pseudoalteromonas* sp. LJ1 (FJ665500.1)	99	-	-	-	-
PM EG14	*Halomonas* sp. Pper-Hx-1972 (EU123940.1)	99	+	+	-	-
PM EG15	*Vibrio* sp. S4639 (FJ457601.1)	99	-	-	-	-
PM EG17	*Pseudoalteromonas* sp. 114Z-11 (GU584139.1)	98	-	-	-	-
PM EG18	*Pseudoalteromonas* *porphyrae* strain HK1 (FJ205736.1)	99	+	+	-	-
PM GO01	*Shewanella* *schlegeliana* (AB081761.1)	99	+	-	-	-
PM GO04	*Vibrio* *splendidus* isolate PB1-10rrnM (EU091337.1)	99	-	-	-	-
PM GO05	*Photobacterium* *kishitanii* strain calba.5.9 (AY642170.1)	99	-	-	-	-
PM GO06	*Shewanella* * schlegeliana* (AB081761.1)	99	+	-	-	-
PM GO08	*Photobacterium* *kishitanii* strain calba.5.9 (AY642170.1)	99	-	-	-	-
PM GO09	*Vibrio* *fischeri* strain SI1E (AY292949.1)	99	-	-	-	NT
PM GO12	*Photobacterium* *kishitanii* strain calba.5.9 (AY642170.1)	99	-	-	-	NT
PM GO13	*Vibrio fischeri* strain SI1Ecomplete (AY292949.1)	99	-	-	-	NT
PM RT05	*Pseudoalteromonas* sp. LJ1 (FJ665500.1)	99	-	+	-	-
PM RT07	*Alteromonas* sp. N98(2010) (HQ188650.1)	99	-	+	-	-
PM RT10	*Vibrio parahaemolyticus* strain NMGB2 (JN561593.1)	99	-	-	-	NT

^a^ NCBI Genbank accession numbers are indicated in brackets; ^b^ NT indicates that the sample was not tested for its ability to produce TTX. The detection limit of TTX using the LC-MS method was 0.1 ng/mL.

**Figure 2:**
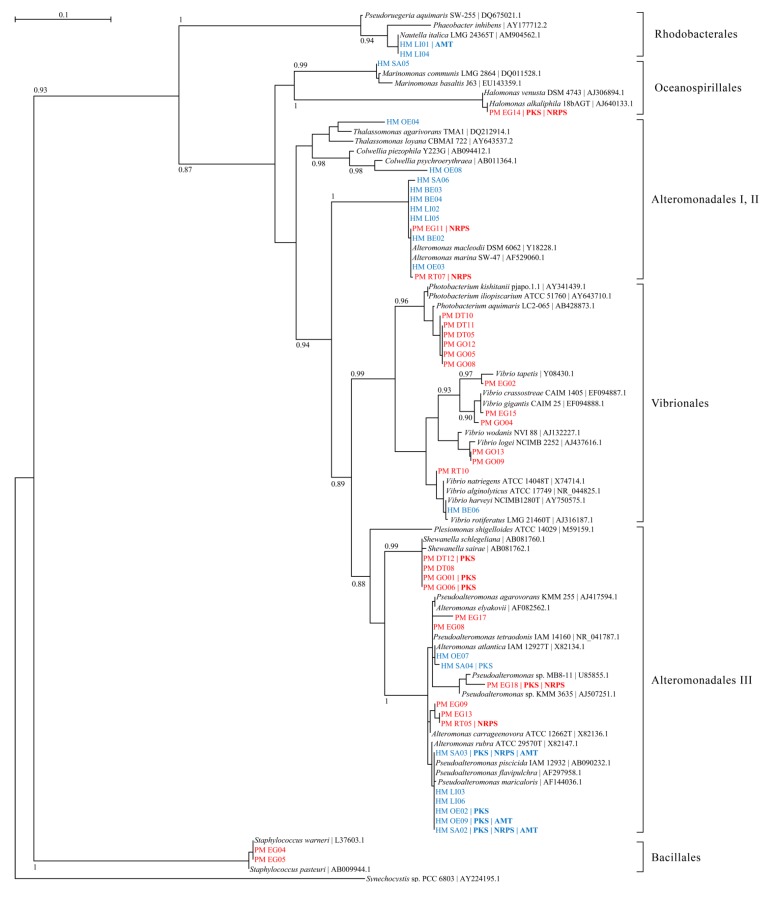
Maximum likelihood phylogenetic tree of 16S rRNA gene sequences of isolates from this study and related bacteria. The *Synechocystis* sp. PCC 6803 16S rRNA gene sequence was used as an outgroup. *Hapalochlaena* sp. sequences (prefix, HM) are indicated with blue text, *P. maculata* sequences (prefix, PM) are indicated with red text. All sequences were submitted to the GenBank database (accession numbers JN618116 to JN618164). Isolates identified as possessing PKS, NRPS, or AMT genes are also indicated.

Phylogenetic analyses revealed that 16S rRNA gene sequences grouped into seven distinct clades representing the orders *Rhodobacterales*, *Oceanospirillales*, *Alteromonadales*, *Vibrionales* and *Bacillales* (Figure 2). In general, *H*. *maculosa* sequences grouped separately from *P*. *maculata* sequences, indicating distinct microbial communities in these two animals. One exception to this was the *P*. *maculata* isolate PM EG11, which grouped phylogenetically with the *Alteromonas* sp. from *H*. *maculosa*, HM BE02 and HM OE03.

*Alteromonadales* isolates grouped into three separate clades and more isolates from the *Alteromonadales* III clade tested positive for biosynthesis genes than in all other clades of isolates. This clade includes the previously reported TTX-producer *Pseudoalteromonas tetraodonis* IAM 14160 [20]. None of the isolates from the *Vibrionales* or *Bacillales* clades possessed complex (NRPS or PKS) biosynthesis genes, as determined by PCR. 

It is well known that specific environmental conditions are critical for the biosynthesis of natural products [21,22] such as TTX [20], as are the synergistic contributions by multiple organisms that lead to substantial increases in metabolite production [23,24]. For these reasons, it is important that the resident microbial communities of TTX-containing organisms are classified and converted to stable laboratory cultures. Microorganisms are the proposed biogenic source of TTX, however, there have been very few studies describing the diversity of bacteria in hosts known to contain TTX. In the past, there been numerous descriptions of culturable TTX-producing bacteria isolated from TTX-containing organisms [5], yet none of these studies reported the entire culturable microbial community. As a result the bacterial diversity in these toxic marine animals has remained largely unknown. In trying to identify a microbial producer of TTX, it is important to understand the entire microbial diversity found within host organisms as it defines the environment where TTX-biosynthesis can occur. This study represents the first study to report the culturable bacterial populations of toxic *H*. *maculosa* and *P*. *maculata*.

The isolation of bacteria from *H. maculosa* and *P. maculata* belonging to seven bacterial genera significantly builds upon the previously published studies investigating TTX-producing bacteria from toxic hosts, the majority of which only describe a single species [5]. Interestingly, two species of molluscs, *Niothaclathrata*, and *Nassarius semiplicatus* were reported [8,25] to possess highly diverse microbial communities, with five bacterial genera identified in each. *Vibrio*, *Tenacibaculum*, *Marinomonas*, *Shewanella*, and *Aeromonas* spp. were identified in *N*. *semiplicatus*, while *Vibrio*, *Pseudomonas*, *Pasteurella*, *Plesiomonas*, and *Aeromonas* spp. were found in *N*. *clathrata*. This is comparable to the diversity in the molluscs used in this study, and suggests that they may have greater bacterial diversity than other TTX-associated hosts. 

The predominance of *Pseudoalteromonas* in both *Hapalochlaena* sp. and *P*. *maculata* suggests that microorganisms of this genus may play a key role in the physiology of their hosts and possibly TTX biosynthesis. To our knowledge, this is the first description of any *Pseudoalteromonas* spp. from molluscs. Furthermore, reports of organ-associated bacteria from molluscs have only described single microorganism associations [26] rather than diverse communities as detailed here.

To date, microbial TTX-producers have been reported from 23 bacterial genera [5,27,28,29,30]. Of these, *Vibrio* spp. have been identified as the TTX-producers in over half the toxic host specimens. The predominance of *Vibrio* spp. is likely due to their abundance in nature and their inherent ability to be cultured from the marine environment. We identified three isolates as *Vibrio* spp. from the two animal hosts. Two of these were isolated from *Hapalochlaena* sp. and one from and *P*. *maculata*, none of which tested positive for TTX (refer to Section 2.2). Unfortunately, useful phylogenetic comparisons were unable to be performed between isolates from this study and those from previous studies of TTX-associated symbionts. Of the twenty-five studies on TTX production by bacteria, only four of these utilized molecular taxonomic methods as part of their bacterial identification procedure. Only two of these studies [8,31] have submitted their data to public databases, hence comparison of 16S rRNA gene sequences from these *Vibro* strains was not possible.

We have shown here that both animals host a diverse, culturable, microbial community. Studies regarding TTX production by bacteria, including the present study, have only focused on pure cultures. When TTX-producing bacteria have been identified, the strains have yielded only low amounts of TTX [8,9] which could not account for the levels of toxin present in the host organisms. 

### 2.2. Overestimation of Abundance and Diversity of TTX-Producing Bacteria

TTX was not detected by liquid chromatography-mass spectrometry (LC-MS) in any of the *H*. *maculosa* organ extracts. TTX was present in the *P*. *maculata* samples with a uniform distribution throughout those organs tested (Table 3). The *P*. *maculata* egg-derived extracts were also found to contain TTX (Table 3). TTX or its analogues were not detected via LC-MS in any of the bacteria-derived methanol extracts tested (Table 1 and Table 2). Bacteria-derived methanol extracts from *N. semiplicatus* isolates were not found to contain TTX or related analogues via LC-MS (Table 4). This is contrary to a previous study [8], which reported these strains as TTX producers, via immunoassay approaches.

**Table 3:** Detection of tetrodotoxin (TTX) in whole-organ homogenates.

Host Organism	Tissue tested	TTX concentration (mg/kg)
*Hapalochlaena* sp.	Posterior salivary gland	BDL ^a^
	Beak	BDL
Pleurobranchaea maculata	Eggs	5.19
	Digestive tract	4.23
	Reproductive tract	3.84
	Gonads	3.54

^a^ BDL indicates that the concentration of TTX was below the detectable limit of the instrumentation. The detection limit of TTX using the LC-MS method was 0.1 ng/mL (equivalent to 3 × 10^−4^ mg/kg of TTX in tissue).

**Table 4:** Screening of *Nassarius semiplicatus* bacterial isolates for putative TTX-biosynthesis genes and a comparison of ELISA and LC-MS methods for the detection of TTX.

Strain ^a^	PCR screening			TTX detection (ELISA)	TTX detection
	PKS	NRPS	AMT	(ng/g) ^b^	(LC-MS)
*Vibrio* sp. 34HU9 (EU268265)	+	+	−	169	BDL ^c^
*Marinomonas* sp. 38JIA1 (EU268259)	−	+	−	98	BDL
*Tenacibaculum* sp. 30ORI8 (EU268261)	−	+	−	54	BDL

^a^ Strains are as described in Wang, X., *et al.*, 2008 [8]; ^b^ ELISA results shown are those previously reported by Wang, X., *et al.* 2008 [8]. The detection limit of TTX using the ELISA method was 5 ng/mL (equivalent to 15 ng/g of TTX in tissue); ^c^ BDL indicates that the concentration of TTX was below the detectable limit of the instrumentation. The detection limit of TTX using the LC-MS method was 0.1 ng/mL (equivalent to 0.3 ng/g of TTX in tissue).

The failure to identify and culture a TTX-producing microbe in this study (Table 1 and Table 2) despite numerous reports of isolations from many other TTX-associated hosts was not completely unexpected. Captivity of the *P. maculata* specimen for 12 days prior to dissection and bacterial isolation may have influenced the microbiota present in our isolation process. Additionally, *P. maculata* have been shown to have high variability in TTX concentrations within individuals [32] suggesting an exogenous source of TTX. Whether this is due to environmental factors or variations in the abundance of bacterial producers is unknown. However, changes in environmental conditions or bacterial diversification may lead to loss of TTX-producing capability. Such a loss of toxin production capability has been reported in other toxin-producers, including the cyanobacteria *Cylindrospermopsis raciborskii*, which produces cylindrospermopsin [33]. These factors may have contributed to the lack of TTX-production by bacteria in this study. Furthermore, it is possible that an unculturable bacteria may be responsible for the production of TTX in these organisms.

Many previous studies have focused exclusively on the detection of TTX in bacterial cells [8,11,25,30]. Therefore, this study did not investigate the presence of TTX in bacterial culture supernatants. Indeed, the presence of TTX at high levels in the supernatant would be represented by a presence of TTX in bacterial cells.

In the many instances where chemical defences are conferred to progeny, the amount of toxin invested into the eggs or larvae is much lower than that of the adult [34]. However, in this and previous studies [7,35], the level of TTX detected in *P*. *maculata* eggs was comparable to that found in the adult. It has also been shown that *P*. *maculata* larvae possess concentrations of TTX equivalent to that of the adult [7]. However, a lack of TTX in the *P*. *maculata* egg-derived bacterial isolates indicates that vertical transmission of TTX-producing bacteria is unlikely in *P*. *maculata* [36]. 

Reported TTX-producers include species from *Vibrio*, *Pseudomonas*, *Alteromonas*, *Marinomonas*, and *Shewanella* genera, and bacteria of the aforementioned genera have been previously isolated from *H*. *maculosa* [37]. Many studies describing the isolation of TTX-producing bacteria from host animals based their findings on the detection of TTX-like activity using non-specific mouse bioassays, or chemically by GC-MS [25,37,38]. Ions characteristic of TTX observed from GC-MS analyses have also been attributed to peptone, a common growth media constituent [39]. Furthermore, the majority of reports that identify TTX in bacteria were not supported by the structure characterization using NMR or MS/MS of a purified compound. This is highlighted in this study where *N. semiplicatus* isolates, which had previously tested positive for TTX using ELISA [8], were not found to produce TTX using more sensitive spectrometric methods. The *N. semiplicatus* specimen from which the bacteria were originally isolated had tested positive for TTX using LC-MS [8] indicating that the bacteria may not be the true producers of TTX in this organism. Taken together, these observations may have contributed to an over-estimation of the number of bacterial TTX producers in the literature. 

### 2.3. Mining Host-Associated Bacteria for Proposed TTX Biosynthesis Genes

Seven isolates from *Hapalochlaena* sp. were found to contain at least one of the targeted biosynthesis genes (Table 1). Two *Pseudoalteromonas* isolates, HM SA2 and HM SA3, were positive for PKS, NRPS, and AMT genes and one *Pseudoalteromonas* isolate, HM OE9, contained both PKS and AMT genes. Furthermore, three *Pseudoalteromonas* isolates contained either a PKS or NRPS genes.

DNA sequencing of PKS and NRPS clone libraries of the *Pseudoalteromonas* isolate HM SA03 revealed four unique PKS ketosynthase domains and six unique NRPS adenylation domains. Many of these domains showed little amino acid similarity (less than 60% identity) to characterized PKS and NRPS domains and are therefore proposed to be novel.

A total of eight *P*. *maculata* isolates contained at least one of the targeted biosynthesis gene types (Table 2). These genes had similarities (70%–97%) to known sequences in the NCBI database, as determined by BLAST analysis. Three *Shewanella* isolates contained only PKS genes, while two *Alteromonas* isolates and a *Pseudoalteromonas* isolate contained only NRPS genes. Single *Halomonas* and *Pseudoalteromonas* strains contained both PKS and NRPS genes. AMT genes were not PCR amplified from any of the *P*. *maculata* isolates. 

All three *N. semiplicatus* isolates contained NRPS genes, however, only the *Vibrio* isolate, HU9, contained PKS genes and none of the *N. semiplicatus* isolates screened contained AMT genes. It is unlikely that TTX is biosynthesized via an NRPS alone [5]. Hence, it is possible that these strains are not true producers of TTX, but may produce a structurally similar compound that may test positive with antibody-based assays and provide a false positive result.

Sequence analysis of AMT PCR products revealed significant similarity to AMT (e.g., c*yrA*) from toxic *Cylindrospermopsis* species [18]. This is significant because CyrA is involved in the biosynthesis of the potent toxin, cylindrospermopsin (Figure 1), which also incorporates an arginine as is proposed for TTX. An alternate biosynthetic route to TTX, which does not require an AMT, but rather, incorporates an intact argininehas been proposed [13,15,16]. However, feeding studies with guanido-^14^C labeled argininecould not confirm the origin of the guanidine moiety in TTX [16], and thus incorporation of intact arginine via an NRPS cannot be discounted. Gene sequences derived from NRPS and PKS PCR were clearly indicative of adenylation and ketosynthase domains, respectively, they did not show significant similarity to any characterized gene clusters and thus cannot be directly linked to the biosynthesis of any known toxin. Therefore, although NRPS and PKS are predicted to be involved in TTX biosynthesis, we cannot confirm the identification of putative TTX biosynthesis genes in this study. Taken together, these findings suggest that AMT are indeed useful targets for PCR-directed screening of microorganisms for toxic alkaloid biosynthesis gene clusters due to their rarity in microorganisms compared to NRPS and PKS-coding genes.

Two isolates from *Hapalochlaena* sp., HM SA02 and HM SA03, derived from the octopus’ salivary glands, and identified as *Pseudoalteromonas* spp., tested positive for all three types of biosynthesis genes. Sequencing of HM SA03-derived clone libraries containing amplified PKS and NRPS gene fragments, revealed unusual genetic diversity, indicative of the potential to biosynthesize small and possibly novel molecules. Additionally, crude chemical extracts derived from the HM SA03 isolate showed moderate inhibitory effects against *Staphylococcus aureus* (data not shown). Though we did not detect TTX or its analogues in the extracts, the isolate is of interest from a small molecule biosynthesis standpoint. Genome sequencing in the future may allow us to ascertain whether this organism possesses a candidate gene cluster for the production of TTX.

## 3. Experimental Section

### 3.1. Specimen Collection

*Hapalochlaena* sp. was purchased from Peter Fearnside (Seafish Aquarium Life), who collected the specimen from Moreton Bay, Australia. This specimen was immediately transported to the laboratory in a plastic bag containing seawater (300 mL). The total time from specimen collection until arrival in the laboratory was approximately 24 h. It was housed in a 10 L aquarium filled with 4 L sterile seawater at 20 °C for 48 h prior to dissection. *Hapalochlaena* sp. was initially identified by the authors as *H. maculosa*, however, an anonymous reviewer has identified the specimen in this study as either *H. fasciata* or *Hapalochlaena* sp. 5.

The *P*. *maculata*specimen was collected from the sediment surface in 2–3 m deep water at Narrow Neck Beach, Auckland, New Zealand. It was placed in a plastic bag containing seawater (300 mL) and transported to the laboratory in a thermo insulated container. The specimen was maintained in a 19 L aquarium filled with 11 L of filtered seawater at 20 °C and aerated using a fish tank pump. It was fed twice weekly on Greenshell™ mussel (*Perna canaliculus*). After 12 days, *P*. *maculata* and its laid egg-mass were removed from the tank prior to dissection.

### 3.2. Specimen Dissection

The *Hapalochlaena* sp. specimen was placed at 4 °C for 2 h prior to dissection. The posterior salivary gland, beak and surrounding soft tissue, digestive glands, ovaries, and eggs were aseptically dissected from the specimen and sterilized (using 70% ethanol (v/v)). Organs were homogenized in 10 mL sterile Milli-Q water (Millipore) using a Janke and Kunkel Ultra Turrax (IKA Laboritechnik) for 10 s at maximum amplitude.

The *P*. *maculata* specimen was placed in ice for 2 h and then dissected using a sterile scalpel. The gonad, digestive tract, and reproductive organs were removed and homogenized. The egg mass was washed several times in 70% ethanol (v/v) and homogenized in 5 mL sterile seawater. A sub-sample of each organ and egg homogenate was frozen for subsequent TTX analysis. 

### 3.3. Bacterial Culturing and Genomic DNA Extraction

To ensure adequate coverage of unique bacterial isolates, 10-fold serial dilutions of organ homogenates were each spread on Marine (Bacto BD), Vaatanen Nine Salts Solution (VNSS), Thiosulfate Citrate Bile Salts Sucrose (TCBS) (Acumedia), and Tryptic Soy Agar (Bacto BD) solid media and incubated at 20 °C. Morphologically unique colonies that were observed on each plate were further sub-cultured until pure cultures were obtained. Bacterial isolates were assigned an identifier using a six-character code. The first two letters indicate the host organism, either *Hapalochlaena* sp. (HM) or *P*. *maculata* (PM). The next two characters indicate the organ from which the bacteria was isolated and its isolate number, either salivary gland (SA), beak (BE), digestive glands (LI), ovary and eggs (OE), egg sac (EG), digestive tract (DT), reproductive tract (RT), or gonads (GO). Genomic DNA was extracted from these isolates using a xanthogenate-SDS protocol as previously described [40]. Approximately 100 mg of cells were resuspended in 500 µL XS buffer (1% potassium ethyl xanthogenate, 800 mM ammonium acetate, 100 mM Tris-HCl pH 7.4, 20 mM EDTA, 1% sodium dodecylsulfate) and inverted several times to mix. The tubes were incubated for 120 min at 65 °C. The tubes were vortexed for 10 s and incubated for a further 60 min at 65 °C. The samples were placed on ice for 10 min, and then centrifuged for 10 min at 12,000 *g* to remove cell debris. The resulting supernatant was washed twice with phenol:chloroform:isoamyl alcohol (25:24:1). DNA was then precipitated with 2 volumes absolute ethanol and centrifuged for 30 min at 14,000 *g*. The resulting DNA pellet was washed with 70% ethanol, centrifuged for 30 min at 14,000 *g* and resuspended in TE buffer (10 mM Tris-HCl, pH 7.4; 1 mM EDTA, pH 8). Three bacterial isolates from *N. semiplicatus* were kindly provided by Rencheng Yu, Institute of Oceanology, Chinese Academy of Sciences [8].

### 3.4. Identification of Bacterial Isolates

PCR amplification of approximately 1400 bp of the 16S rRNA gene was performed using the bacterial-specific primers 27fl and 1494rc [41]. Each PCR reaction mixture contained 2.5 mM MgCl_2_, 0.15 mM each dNTP, 10 pmol each primer, 0.2 U of Bio*Taq* DNA polymerase, the appropriate PCR buffer (Bioline) and approximately 1 ng DNA. Thermal cycling conditions were as follows: initial denaturation at 94 °C for 2 min, 35 cycles of denaturation at 94 °C for 30 s, annealing at 55 °C for 30 s, extension at 72 °C for 1 min, followed by a final extension step at 72 °C for 7 min. PCR amplification products were electrophoresed through a 1% agarose gel, visualized by staining with 0.5 μg·mL^−1^ ethidium bromide, and documented with a Gel Doc XR camera using Quantity One 4.6.1 software (BioRad). PCR amplicons were precipitated for 15 min using 2 volumes ice-cold 95% ethanol and 1/10 volumes 3M sodium acetate. Precipitated DNA was washed with 70% ethanol and resuspended in Milli-Q water.

Automated sequencing reactions were performed using approximately 50ng of purified PCR product and 3.2pmol of primer (26fl/1494rc) using the Prism Big Dye cycle-sequencing system and ABI 3730 DNA analyser sequencer (Applied Biosystem). Isolates with identical nucleotide sequences considered to be the same and a single representative was selected for downstream analysis. Closest 16S rRNA gene homologies were identified using the BLAST search program (NCBI). 

### 3.5. PCR Screening of Bacterial Isolates for Biosynthesis Genes

All isolates were screened for the presence of PKS genes using the degenerate primer set DKF/DKR (Table 5) [42], NRPS genes using the degenerate primer set MTF2/MTR2 [43] and AMT genes using the degenerate primer set ATFI/ATRI [44]. Expected sizes for PCR amplification products were 700 bp for PKS and AMT, and 1000 bp for NRPS products. PCR reactions were carried out as described above, except 25 pmol of each primer was used. Thermal cycling conditions were as followed: initial denaturation at 92 °C for 2 min, 35 cycles of denaturation at 92 °C for 10 s, annealing as outlined in Table 5 for 30 s, extension at 72 °C for 1 min, followed by a final extension step at 72 °C for 7 min. Purification of PCR amplification products, DNA sequencing and gene identification using the BLAST server was performed as described above.

**Table 5:** Primers used for identification and screening of bacterial isolates.

Primer	Sequence	Tm^a^	AT^b^	Reference
27fl	AGAGTTTGATCCTGGCTCAG	61	55	[41]
1494rc	TACGGCTACCTTGTTACGAC	59	55	[41]
DKF	GTGCCGGTNCC(A/G)TGNG(T/C)(T/C)TC	67	55	[42]
DKR	GCGATGGA(T/C)CCNCA(A/G)CA(A/G)(C/A)G	65	55	[42]
MTF2	GCNGG(C/T)GG(C/T)GCNTA(C/T)GTNCC	64	52	[43]
MTR2	CCNCG(A/G/T)AT(T/C)TTNAC(T/C)TG	47	52	[43]
ATfwd1	GT(A/C/G)TG(T/C)CC(A/T)(A/C)G(G/C)GA(T/C)GT(A/C/G)ATG	57	55	[44]
ATrev1	AT(A/G)TCCCA(A/T)(A/G)T(C/G/T)C(A/G)CA(A/G)TG	62	55	[44]

^a ^Tm is the theoretical melting temperature of the PCR primers, given in °C; ^b ^AT is the annealing temperature used in PCRs containing these primers, given in °C.

PCR screening of *H*. *maculosa* isolate HM SA03 revealed multiple NRPS and PKS genes, hence cloning of these genes was performed. PKS and NRPS PCR amplification products were gel-purified and ligated into pCR2.1-TOPOvector using a TOPO-TA Cloning kit following protocols provided by the manufacturer (Invitrogen) and transformed into chemically competent *Escherichia coli* DH5α. Ten clones from each clone library were randomly selected and PCR amplified using vector-directed primers (MpF and MpR). Purification, sequencing and analysis of PCR products were performed as described above.

### 3.6. Phylogenetic Analysis of 16S rRNA Gene Sequences

Partial sequences of 16S rRNA genes obtained from this study, as well as 42 reference sequences obtained from NCBI Genbank, were aligned using Muscle [45]. A phylogenetic tree was constructed from a 539 bp alignment with maximum likelihood algorithm, PhyML [46], using the general time reversible with gamma distribution (GTR + G) base substitution model determined using the web-based application, FindModel [47]. The tree was rooted with *Synechocystis* sp. PCC 6803 (Genbank accession number AY224195.1) as an outgroup. The aLRT SH-like approach was used as an estimate of branch reliability [48].

### 3.7. Toxin Extraction from Organ Homogenates and Bacterial Isolates

Sub-samples (1 g, wet weight) of each organ homogenate were extracted with 3 volumes (approximately 3 mL) of HPLC-grade methanol (0.1% v/v acetic acid) and centrifuged (3000× *g*, 10 min) to remove cell debris. 

Cultured organisms that possessed PKS genes, as determined by PCR amplification, were grown in 1 L volumes, as were bacteria isolated from *P*. *maculata*eggs, to observe whether TTX was produced in culture. The cultures were grown aerobically at 20 °C for 3 days, with shaking in peptone (0.5%; Oxoid) in filtered seawater. This was used as a culture media as it has been shown to promote the production of TTX in bacteria [20]. Cultures were centrifuged (3000× *g*, 10 min) and the supernatant removed. Cell pellets were then extracted as described for organ homogenates. *N. semiplicatus* bacterial isolates were grown and chemically extracted using conditions described previously [8]. 

### 3.8. Analysis of Extracts by Liquid Chromatography-Mass Spectrometry

To determine whether TTX was present in methanol extracts prepared from the organs and associated microbes of *P*. *maculata*, LC-MS was performed as previously described [7]. Extracts of *Hapalochlaena* sp. and its associated bacterial isolates were analysed by LC-MS using a Thermo Finnigan Surveyor HPLC and autosampler equipped with a Thermo Finnigan LCQ Deca XP Plus fitted with an electrospray source. Separation of analytes was obtained on a Phenomenex Luna 3 μm C18 column (2.1 mm × 150 mm) at a flow rate of 100 μL·min^−1^. After an initial period of 10 min using a solvent gradient of 95% water (10 mM heptafluorobutyric acid), the mixture was ramped to 100% acetonitrile over 30 min.

To determine if the bacterial pellet matrix resulted in any suppression or enhancement of the TTX signal during LC-MS analysis a spiked recovery experiment was undertaken. Samples were spiked in duplicate with pure TTX (Tocris Bioscience, Cat. No: 1078) to give final concentrations of 1 ng/mL, 2 ng/mL, 10 ng/mL and 100 ng/mL. No matrix effects were measured.

## 4. Conclusions

This study has provided a better understanding of the microbial diversity in two TTX-associated molluscs and revealed the potential for the production of PKS and NRPS-derived natural products from their cultured bacteria. We set out to isolate culturable microorganisms with the aim of positively identifying a TTX producer. Despite our efforts we are yet to identify such an organism although a number of candidates have been selected for further investigation of their natural products. It is plausible that the TTX producing organism may not be easily cultured under standard laboratory conditions, as is the case with the majority of all bacteria [49]. It is also possible that we may well have isolated a microbe with the genetic potential to biosynthesize TTX but production was not achievable *ex situ*. Biosynthetic precedents of structurally similar compounds suggest an NRPS or PKS origin for TTX, however, there is no experimental evidence to support this. It is plausible thatthe highly unusual structure of TTX may be the product of an unusualpathway utilizing novel enzymes. Characterization of the culturable microbial communities of *Hapalochlaena* sp. and *P*. *maculata* indicated a greater than anticipated level of diversity. It is reasonable to presume that an unculturable, or yet-to-be cultured, TTX-producer exists. To this end a metagenomics-based approach to study the entire communities of these molluscs is in progress. This will not only extend our knowledge of the microbial community, but also provide us with clues as to how to culture members of the community in the laboratory, and aid the search for the elusive TTX producer and its biosynthetic pathway.
